# Physics-Aware Generative Demasking: Spatially Conditioned Diffusion for Robust Transient Detection in Industrial Noise

**DOI:** 10.3390/e28040364

**Published:** 2026-03-24

**Authors:** Hailin Cao, Zixi Lv, Jinjie Hu, Hui Wang, Lisheng Yang, Guoxin Zhang

**Affiliations:** 1 School of Microelectronics and Communication Engineering, Chongqing University, Chongqing 400044, China; hailincao@cqu.edu.cn (H.C.); 20192467@cqu.edu.cn (Z.L.);; 2 Great Wall Motor Company Limited, Chongqing Branch, No. 666, Fenglong Avenue, Yongchuan District, Chongqing 402100, China; bdzgx@163.com

**Keywords:** transient acoustic monitoring, acoustic event detection, conditional diffusion model, random convolution kernels, restore-then-classify

## Abstract

Detecting transient “click” sounds during connector insertion is pivotal for automotive assembly quality but remains intractable due to high-intensity, non-stationary industrial noise. This paper introduces a physics-aware generative demasking framework that integrates acoustic spatial priors with conditional diffusion modeling. We propose the spatially conditioned diffusion probabilistic model (SC-DPM), where an ambient reference signal acts as a physical constraint to steer the reverse diffusion process. By exploiting the spatial decay of insertion sounds, this mechanism effectively disentangles the target transient from the background noise manifold, reconstructing high-fidelity spectro-temporal features. Discriminative temporal patterns are extracted using causal random convolutional kernels with causal dilations and local proportion of positive values (LPPV) pooling. Experiments on real-world datasets demonstrate 93.3% accuracy. The proposed “restore-then-classify” paradigm significantly enhances robustness against acoustic variability, establishing a scalable methodology for precise industrial monitoring under extreme noise conditions.

## 1. Introduction

In the era of Industry 4.0, zero-defect assembly is a paramount objective for automotive manufacturing. Specifically, the reliability of electrical connector insertion is critical; partial or improper mating can lead to intermittent signal failures, posing severe safety risks and incurring high warranty costs. While traditional verification methods such as manual “push-pull” checks and post-process electrical continuity testing [1] have been widely utilized, they are inherently invasive and interrupt the continuous production workflow. Manual inspections are labor-intensive and susceptible to human fatigue, while continuity testing requires a powered system and assumes that all mechanical faults manifest immediately as electrical open circuits. Consequently, these methods often fail to meet the stringent cycle time (Takt time) requirements of modern high-throughput assembly lines, creating an urgent demand for in situ, noninvasive process monitoring.

Among noninvasive modalities, acoustic event detection (AED) has emerged as a promising solution, particularly for monitoring the characteristic “click” sound that signifies a secure mechanical lock. Unlike computer vision (CV) systems, which frequently suffer from visual occlusion due to the operator’s hands or complex cabling, acoustic sensing is omnidirectional and unaffected by line-of-sight constraints. Advancements in deep architectures, such as convolutional neural networks (CNNs) [2] and convolutional recurrent neural networks (CRNNs) [3], have significantly improved the capability to model temporal and spectral characteristics. Furthermore, large-scale pretrained models like PANNs [4] have demonstrated robust generalization.

However, deploying AED in real-world factories presents a formidable scientific challenge: the extreme signal-to-noise ratio (SNR) paradox. The target “click” is a short-duration (transient) signal with subtle morphological features, while the background is dominated by high-intensity, non-stationary industrial noise. As characterized in recent industrial noise management studies [5], such environments are replete with impulsive interference (e.g., pneumatic tools) and dynamic machinery sounds that defy simple statistical modeling. Conventional signal processing techniques, such as frequency-dynamic convolutions [6,7], often degrade the target transient while attempting to suppress this complex noise. Similarly, advanced data augmentation strategies [8,9] or recent universal audio language models like Qwen-Audio [10] and separation methods like AudioSep [11,12], often struggle to generalize across the highly fluctuating acoustic environments typical of automotive workshops without incurring prohibitive computational costs.

To overcome the limitations of discriminative models, denoising diffusion probabilistic models (DDPMs) [13] have recently emerged as a transformative paradigm. This framework was further refined by Nichol and Dhariwal [14] for improved sampling efficiency, while Turner et al. [15] provided a unified theoretical perspective on the diffusion process. Unlike generative adversarial networks (GANs) [16,17], which are prone to mode collapse and training instability, diffusion models learn to progressively reconstruct the signal structure from Gaussian noise through a stable stochastic process. Studies such as DiffWave [18], WaveGrad [19], and AudioLDM [20] have demonstrated that diffusion models can recover fine-grained waveform details theoretically ideal for restoring “buried” industrial signals.

Despite their generative power, applying standard diffusion models to industrial blind source separation remains problematic because they remain “spatially blind”. They struggle to distinguish between the “foreground” transient and the “background” interference without explicit guidance, often hallucinating noise as part of the signal. Furthermore, the iterative reverse sampling process incurs high computational costs, which seemingly contradicts the real-time requirements of factory monitoring. This reveals a critical gap: the lack of physical constraints in current generative frameworks.

Recognizing that insertion sounds decay rapidly over distance while ambient noise remains diffuse, we propose leveraging this physical spatial asymmetry as a hard constraint. Inspired by the differential mechanisms in the differential transformer [21], which effectively suppress noise by leveraging contextual differences, we present a physics-aware generative demasking framework. Specifically, we propose the spatially conditioned diffusion probabilistic model (SC-DPM), which injects the ambient reference signal as a dynamic condition into the reverse diffusion process. To reconcile the computational overhead of the generative front-end with real-time demands, we integrate a lightweight feature extraction mechanism based on causal random convolutional kernels (SeeROCKET) [22], ensuring the high-fidelity reconstruction is translated into rapid, robust decision making.

The key contributions of this work are as follows:

1. We propose a physics-aware acquisition framework utilizing a wearable dual-sensor set-up. By contrasting audio characteristics captured at the source (glove) and the periphery (chest/desk), we construct a hard spatial constraint that effectively disentangles the target transient signatures from the complex background noise manifold, utilizing the ambient signal as a dynamic reference for the environmental noise floor.
2. We develop a spatially conditioned diffusion probabilistic model (SC-DPM) for signal enhancement. By injecting the ambient noise reference as a dynamic guidance condition into the reverse diffusion process, the model learns to physically demask the target signal. Unlike traditional subtractive methods, this approach leverages generative modeling to restore high-fidelity morphological details of the connector insertion sound from heavily corrupted mixtures.
3. We introduce an efficient feature extraction mechanism based on causal random convolutional kernels. By employing causal dilations and local proportion of positive values (LPPV) pooling, this method captures the transient morphological features of the enhanced “click” sound without computational overhead, facilitating rapid and robust detection suitable for real-time industrial cycles.

## 2. Related Work

### 2.1. Time Series Classification with Random Convolution Kernels

Time series classification (TSC) aims to assign class labels to sequential data points recorded at regular intervals. Formally, a time series *X* can be represented as $X=\{x_{1},x_{2},\ldots ,x_{T}\}$, where $x_{t}$ represents the value of the time series at time step *t* and *T* denotes the total length. While deep learning approaches have gained popularity, kernel-based methods utilizing random convolutions have established a new state of the art due to their efficiency and scalability.

The Random Convolutional Kernel Transform (ROCKET) framework introduced the paradigm of transforming time series into feature vectors using a vast number of random convolutional kernels, subsequently training a linear classifier. By employing pooling operators such as proportion of positive values (PPV) and global maximum pooling (GMP), ROCKET achieves exceptional accuracy with a fraction of the computational cost of deep neural networks [23].

Building on this foundation, several variants have been proposed to optimize feature extraction. MiniRocket streamlines the process by adopting a deterministic approach with fixed kernel parameters and relying exclusively on PPV pooling. This modification significantly reduces computational overhead while maintaining accuracy suitable for large-scale applications [24]. MultiRocket further extends this architecture by incorporating first-order difference transformations and a diverse set of pooling operators—including mean of positive values (MPV), mean of indices of positive values (MIPV), and longest stretch of positive values (LSPV)—to capture more complex dynamic patterns and transform characteristics [25].

To address specific resource constraints in embedded environments, evolutionary and pruning strategies have been introduced. S-ROCKET utilizes population-based optimization to select a compact subset of informative kernels that maximize classification accuracy while minimizing computational costs [26]. Similarly, P-ROCKET improves upon this by incorporating dynamic regularization penalties to accelerate inference without compromising performance [27]. Furthermore, SelF-Rocket introduces a dynamic feature selection mechanism that adaptively identifies the optimal combination of input representations and pooling operators, ensuring state-of-the-art accuracy with reduced redundancy tailored to the specific dataset [28,29].

Parallel to these kernel methods, simple pooling front-ends (SIMPFs) have also been explored for efficient audio classification, demonstrating that simple aggregation operations can sometimes rival complex learnable front-ends [30]. However, standard ROCKET variants and global pooling methods tend to obscure local temporal details critical for detecting short-duration transient events. To mitigate this, SeeROCKET [22] proposes a forecasting-oriented architecture based on causal convolutions and the local proportion of positive values (LPPV). By enforcing causality (ensuring operations depend only on past data) and preserving local temporal dynamics through segmented pooling, SeeROCKET is uniquely suited for real-time sound event detection tasks where latency and transient preservation are paramount.

### 2.2. Denoising Diffusion Models and Physics Awareness

Denoising diffusion probabilistic models (DDPMs) have emerged as the dominant generative framework for modeling complex data distributions, superseding generative adversarial networks (GANs). Ho et al. [13] established the foundational architecture, utilizing a parameterized Markov chain to reconstruct data from Gaussian noise. This framework was subsequently refined by Nichol and Dhariwal [14] to enhance sampling efficiency and learn variances, while Turner et al. [15] recently provided a unified theoretical perspective on the diffusion process. Unlike conditional GANs, which have demonstrated efficacy in specific spatial localization tasks such as fast radio burst (FRB) detection with cluster-fed telescopes [31] but often suffer from mode collapse and training instability, diffusion models offer a stable, likelihood-based objective, ensuring high-fidelity signal reconstruction.

In the audio domain, conditional diffusion probabilistic models (CDPMs) have been extensively applied to speech enhancement [32], acting as robust nonlinear filters. However, industrial acoustic environments present unique challenges distinct from speech denoising, characterized by non-stationary machine noise. To address complex environmental interference, insights from broader signal processing fields are valuable. For instance, in large-scale antenna arrays, location-aware deep learning frameworks [33] and reinforcement learning-based reconfigurable intelligent surfaces [34] have been successfully deployed to achieve degree-of-freedom interference suppression. These advancements highlight a cross-domain consensus: leveraging spatial constraints is essential for handling complex interference.

Recent advancements in generative restoration have further validated the use of priors for signal recovery. Shi et al. [35] introduced Resfusion, a diffusion framework that leverages prior residual noise to guide image restoration, effectively bridging the gap between deterministic degradation models and generative priors. Tailoring these concepts to mechanical sounds, Su et al. [36] proposed a physics-driven diffusion model for impact sound synthesis, accurately modeling the rapid attack and decay envelopes of mechanical interactions. Furthermore, in industrial fault diagnosis, physics-informed frameworks like DiffPhysiNet [37] and implicit models (DDIMs) [38] have proven effective in reconstructing impulsive fault signatures from noisy vibration signals under varying load conditions. The versatility of this approach is further evidenced by applications in biomedical signals, such as photoplethysmography (PPG) denoising [39], and open-domain target sound extraction methods like SoloAudio [40], which utilize semantic cues for isolation.

In this work, we synthesize these advancements into a cohesive framework. Inspired by the residual noise priors in Resfusion [35] and the spatial interference suppression in array processing [33], we propose a spatially conditioned diffusion probabilistic model (SC-DPM). Departing from text-guided methods, we utilize the ambient reference signal as a dense physical condition—effectively a “spatial residual prior”—to guide the precise demasking of connector insertion clicks in complex acoustic environments.

## 3. Methods

### 3.1. System Overview

**Figure 1:**
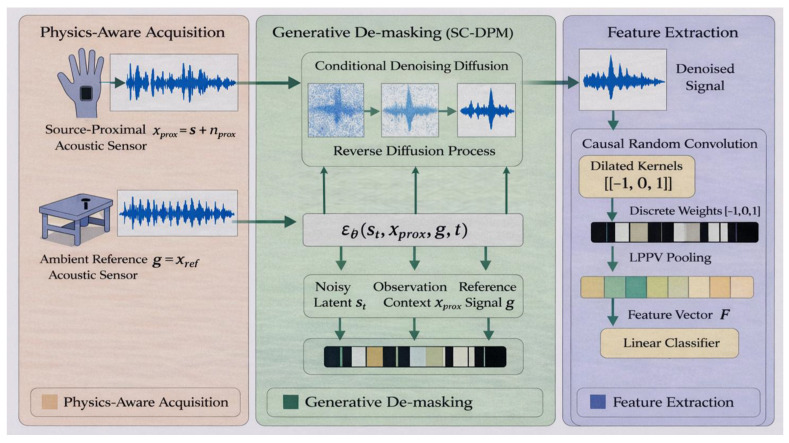
Overview of the proposed physics-aware generative demasking framework. A dual-microphone spatial constraint is integrated with a conditional diffusion model to restore transient signals prior to classification.

The proposed physics-aware generative demasking framework is illustrated in Figure 1. To address the extreme signal-to-noise ratio (SNR) conditions encountered in industrial connector assembly, the system operates through three strictly sequential stages:

- Physics-Aware Acquisition: A dual-sensor configuration is employed to simultaneously capture a source-proximal acoustic signal and an ambient noise reference, thereby imposing a physical spatial constraint on the observed mixture.
- Generative Demasking (SC-DPM): A spatially conditioned diffusion probabilistic model (SC-DPM) leverages the ambient reference as an explicit conditioning variable to disentangle and reconstruct the clean insertion transient from the noisy observation.
- Causal Feature Extraction: The reconstructed waveforms are transformed into discriminative representations using causal random convolutional kernels, enabling temporally precise and computationally efficient classification.

### 3.2. Physics-Aware Acquisition Strategy

In industrial assembly environments, the acoustic signature of connector insertion typically manifests as a short-duration, high-frequency impulsive transient. According to the inverse square law governing spherical wave propagation, the energy of such near-field transients decays rapidly with distance. In contrast, background industrial noise—originating from pneumatic tools, robotic actuators, or conveyor systems—can be approximated as a far-field, spatially diffuse, and temporally persistent process.

This inherent spatial asymmetry is exploited to construct a spatial feature differencing (SFD) acquisition strategy. Specifically, two microphones with distinct spatial roles are deployed:

- Source-Proximal Sensor (x): This is mounted on the operator’s glove at a distance of approximately 10cm from the connector. This sensor captures the high-energy insertion transient while remaining contaminated by local environmental noise.
- Ambient Reference Sensor (g): This is positioned on the operator’s chest or a nearby workbench at a distance exceeding 0.3m. Due to distance attenuation and partial body shadowing, this sensor predominantly records environmental noise, with negligible contribution from the insertion transient.

The recorded signals (in discrete vector form $\in R^{L}$) can be modeled as follows:

(1) $$x=s_{0}+n_{\mathrm{prox}},\;g\approx n_{\mathrm{ref}},$$

where $s_{0}$ denotes the clean insertion signal and $n_{\mathrm{prox}}$ and $n_{\mathrm{ref}}$ represent the background noise observed at the two spatial locations. Although these noise components are not identical, they exhibit strong spectral and temporal correlation.

Unlike conventional differential microphone arrays that rely on precise phase alignment, the proposed framework treats the reference signal g as a latent conditioning variable. This design allows the subsequent generative model to learn a nonlinear mapping between $n_{\mathrm{ref}}$ and $n_{\mathrm{prox}}$, enabling effective demasking of the target transient without amplifying phase mismatch errors.

### 3.3. Spatially Conditioned Diffusion Probabilistic Model (SC-DPM)

To reconstruct high-fidelity insertion waveforms under extremely low SNR conditions, we propose the SC-DPM. Unlike standard diffusion models that generate samples unconditionally, our model learns a conditional distribution $p_{\theta }\left(s_{0}|x,g\right)$, where the generation process is guided by both the noisy observation and the spatial noise reference. In this work, the ambient reference g is used as a waveform-level conditioning signal.

#### 3.3.1. Forward Diffusion Process

We define a fixed forward diffusion process solely on the target signal $s_{0}$. Gaussian noise is progressively injected over *T* steps:

(2) $$q\left(s_{t}|s_{t-1}\right)=N\left(s_{t};\sqrt{\alpha_{t}}s_{t-1},\left(1-\alpha_{t}\right)I\right),$$

where ${\left\{\alpha_{t}\right\}}_{t=1}^{T}$ is a predefined variance schedule. The marginal distribution at an arbitrary timestep *t* is

(3) $$q\left(s_{t}|s_{0}\right)=N\left(s_{t};\sqrt{{\bar{\alpha }}_{t}}s_{0},\left(1-{\bar{\alpha }}_{t}\right)I\right),$$

where ${\bar{\alpha }}_{t}=\prod_{i=1}^{t}\alpha_{i}$. As T→∞, $s_{T}$ approaches a standard Gaussian N(0,I).

#### 3.3.2. Conditional Reverse Process

The reverse process reconstructs $s_{0}$ from $s_{T}$, conditioned on the proximal observation x and the ambient reference g. The joint transition probability is parameterized as follows:

(4) $$p_{\theta }\left(s_{t-1}|s_{t},x,g\right)=N\left(s_{t-1};\mu_{\theta }\left(s_{t},x,g,t\right),\sigma_{t}^{2}I\right).$$

We employ a noise prediction network $\epsilon_{\theta }$ to estimate the noise component at step *t*. The reverse mean is derived using the conditional DDPM formulation

(5) $$\mu_{\theta }\left(s_{t},x,g,t\right)=\frac{1}{\sqrt{\alpha_{t}}}\left(s_{t}-\frac{1-\alpha_{t}}{\sqrt{1-{\bar{\alpha }}_{t}}}\epsilon_{\theta }\left(s_{t},x,g,t\right)\right).$$

In implementation, the noisy latent signal $s_{t}$, the proximal observation x, and the ambient reference g are concatenated along the channel dimension and jointly fed into the noise prediction network at each reverse step. The ambient reference is incorporated as a synchronized signal-domain condition. Different from standard conditional diffusion, where the condition is often given as a label or an abstract embedding, the proposed SC-DPM conditions the reverse process on a spatial reference waveform recorded by a separate microphone.

#### 3.3.3. Training Objective

The network is trained to predict the noise residual ϵ using the standard simplified noise prediction objective:

(6) $$L_{\mathrm{simple}}=E_{t,s_{0},\epsilon ,x,g}\left[{∥\epsilon -\epsilon_{\theta }\left(\sqrt{{\bar{\alpha }}_{t}}s_{0}+\sqrt{1-{\bar{\alpha }}_{t}}\epsilon ,x,g,t\right)∥}^{2}\right].$$

This objective forces the model to learn a denoising function that is consistent with the spatial constraints provided by the dual-sensor set-up.

### 3.4. Efficient Feature Extraction via Causal Random Kernels

Following waveform reconstruction, a causal random kernel transformation is applied to extract discriminative features from the restored signal while satisfying real-time processing constraints.

#### 3.4.1. Causal Dilated Convolution

Let $\hat{s}\in R^{L}$ denote the enhanced time-domain signal output by the SC-DPM. A set of *K* random convolutional kernels is generated, each characterized by the weights $w^{k}$, bias $b^{k}$, and dilation factor $d^{k}$. To enforce strict causality, only past and present samples are used:

(7) $$z_{t}^{k}=\sum_{j=0}^{L_{k}-1}w_{j+1}^{k}\cdot {\hat{s}}_{t-j\cdot d^{k}}+b^{k},$$

where kernel lengths $L_{k}\in \left\{7,9,11\right\}$ and exponentially sampled dilations capture temporal structures at multiple scales.

#### 3.4.2. Discrete Kernel Weights

To reduce computational overhead, kernel weights are restricted to the discrete set W={−1,0,1} with uniform probability

(8) $$P\left(w_{j}^{k}=v\right)=\left\{\begin{array}{cc} 1/3, & v\in \{-1,0,1\},\\ 0, & \mathrm{otherwise}. \end{array}\right.$$

This constraint enables efficient implementation using integer addition and subtraction, effectively highlighting high-frequency transient components in the demasked signal.

#### 3.4.3. Local Proportion of Positive Values

To preserve short-duration events that may be obscured by global pooling, the convolution outputs are segmented into *S* local intervals. For the *s*th segment, the local proportion of positive values (LPPV) feature is defined as follows:

(9) $$f_{k,s}=\frac{1}{|I_{s}|}\sum_{t\in I_{s}}I\left(z_{t}^{k}>0\right),$$

yielding a feature matrix $F\in R^{K\times S}$. This representation captures the temporal evolution of transient activity and serves as input to a lightweight linear classifier for final decision making.

## 4. Data Preparation and Experiments

### 4.1. Data Acquisition System

To empirically validate the robustness of the proposed physics-aware generative demasking framework under realistic industrial conditions, we established a synchronized multi-channel data acquisition system and conducted extensive field recordings.

**Figure 2:**
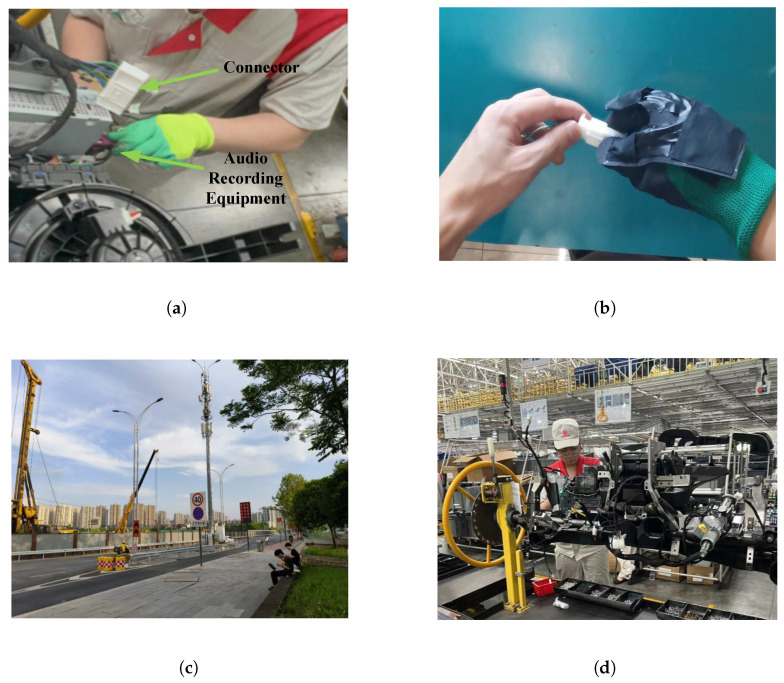
Data acquisition set-up and recording environments. (**a**) The glove-integrated proximal sensor ($x_{\mathrm{prox}}$). (**b**–**d**) Representative scenarios ranging from the quiet laboratory baseline to complex industrial workshops.

The hardware configuration and recording scenarios are illustrated in Figure 2. The sensing set-up strictly implements the physics-aware acquisition strategy defined in Section 3:

- Source-Proximal Sensor ($x_{\mathrm{prox}}$): A high-fidelity electret microphone is integrated into an industrial safety glove, positioned approximately 5cm from the operator’s fingertips (Figure 2a). This sensor is configured to capture the high-intensity near-field transient signature of connector insertions.
- Ambient Reference Sensor ($x_{\mathrm{ref}}$): An auxiliary microphone is deployed in the far field to capture the environmental noise profile correlated with the proximal sensor, providing the necessary spatial condition for the SC-DPM.

For consistency with the notation introduced in Section 3.2, the source-proximal sensor $x_{\mathrm{prox}}$ here corresponds to the proximal observation channel x, while the ambient reference sensor $x_{\mathrm{ref}}$ corresponds to the reference channel g. These terms describe the same sensing channels from the perspectives of physical deployment and model construction, respectively.

To ensure the efficacy of the spatial differencing algorithm, all channels were strictly synchronized and digitized at a sampling rate of $f_{s}$ = 44,100 Hz. This high sampling bandwidth is critical for preserving the high-frequency spectral components (up to 8 kHz) inherent to the impulsive “click” signal.

Data collection spanned three distinct acoustic environments characterized by increasing complexity:

1. Controlled Baseline (Lab): An anechoic setting was used to acquire high-quality ground truth signals of connector insertions, serving as the clean reference.
2. Unseen Non-Stationary Environments (OOD): Recordings were conducted in outdoor construction sites and windy areas. These scenarios introduced irregular, impulsive interference (e.g., impact sounds and wind gusts) to test the model’s generalization capability against out-of-distribution (OOD) noise.
3. Target Operational Environment (Workshop): Field recordings were acquired from an active automotive assembly line. This environment featured continuous, broadband background noise generated by pneumatic tools, automated conveyors, and overlapping human speech.

### 4.2. LLM-Assisted Automated Annotation Pipeline

**Figure 3:**
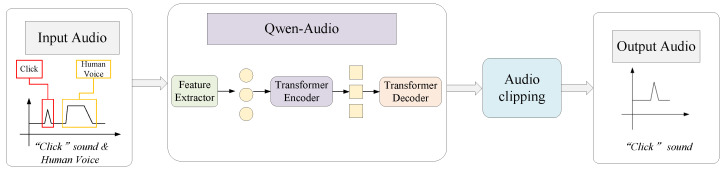
Schematic of the automated annotation pipeline. The system employs a large audio model (Qwen-Audio) to detect post-action voice cues and retroactively segments the synchronized multi-channel audio to isolate the insertion event.

Annotating micro-events in long-duration industrial recordings is notoriously labor-intensive and prone to human error. To facilitate the construction of a large-scale, time-aligned dataset, we developed an LLM-assisted automated data curation pipeline driven by operator voice cues, as shown in Figure 3.

The pipeline converts verbal confirmations into precise temporal labels through a two-stage process:

- Stage I: Voice-Anchored Temporal Localization. To overcome the limitations of traditional energy-based detection in high-noise environments, we employed the pretrained Qwen-Audio model [10]. As a state-of-the-art large audio model (LAM), Qwen-Audio demonstrates exceptional zero-shot generalization in speech understanding amidst noise. We fine-tuned the model for keyword spotting (KWS) to robustly detect specific vocal triggers (e.g., shouting “One”) and pinpoint the timestamp $t_{\mathrm{cue}}$ within the proximal stream.
- Stage II: Synchronized Retroactive Extraction. Leveraging the causal relationship between the physical action and the subsequent vocalization, the system extracts a fixed temporal window $\left[t_{\mathrm{cue}}-2\;s,\;t_{\mathrm{cue}}\right]$ across all synchronized channels. This window is empirically calibrated to encompass the complete action sequence—approach, insertion click, and release—while excluding the vocal cue itself.

This strategy significantly reduced the cost of dataset construction, enabling the rapid generation of labeled samples directly from field operations.

### 4.3. Dataset Organization and Spectral Characteristics

Utilizing the samples harvested via the automated pipeline, we constructed two primary datasets. Crucially, this study relied exclusively on authentic field recordings, preserving the complex acoustic coupling and reverberation characteristics often lost in synthetic mixtures.

- OOD-Driven Training Set (Training): This dataset comprised recordings acquired in diverse non-workshop environments (e.g., construction sites). By training on these harsh, irregular noise conditions, we forced the SC-DPM to learn generalized physical demasking rules rather than overfitting to specific workshop frequency patterns. This strategy enhanced robustness against out-of-distribution (OOD) acoustic shifts.
- In-Domain Evaluation Set (Testing): This dataset consisted exclusively of recordings collected from the actual automotive assembly line. It represents the target domain, characterized by stationary mechanical hum and pneumatic tool transients and serving as the rigorous standard for evaluating the system’s practical performance.

The complete dataset consisted of approximately 11,200 segmented samples. During the training phase, the positive and negative samples were balanced to reduce class bias and prevent overfitting in the presence of noise in industrial environments.

**Figure 4:**
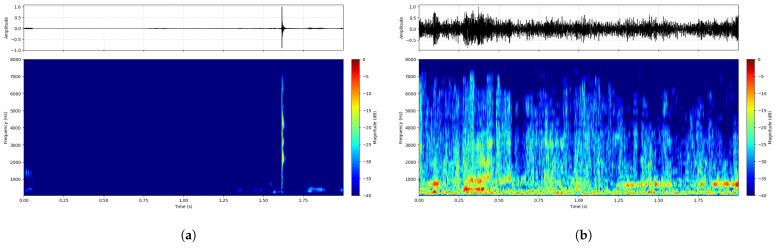
Spectro-temporal analysis of real-world recordings. (**a**) A pristine connector insertion signature captured in the controlled baseline set-up. (**b**) The same type of insertion event captured in the noisy workshop, where the broadband transient is severely obscured by industrial background noise (spectro-temporal masking).

The challenge of detecting signals in these naturally noisy environments is visualized in Figure 4. The spectrograms contrast a connector click captured in the quiet baseline environment against one from the workshop. In the clean domain, the “click” manifests as a distinct broadband vertical transient. In contrast, under real workshop conditions, this signature is severely masked by low-frequency mechanical hum and overlapping wideband noise. Unlike synthetic Gaussian noise, real-world environmental interference exhibits complex non-stationary variations, necessitating the proposed diffusion-based enhancement framework.

## 5. Results and Discussion

### 5.1. Signal Reconstruction Analysis

**Figure 5:**
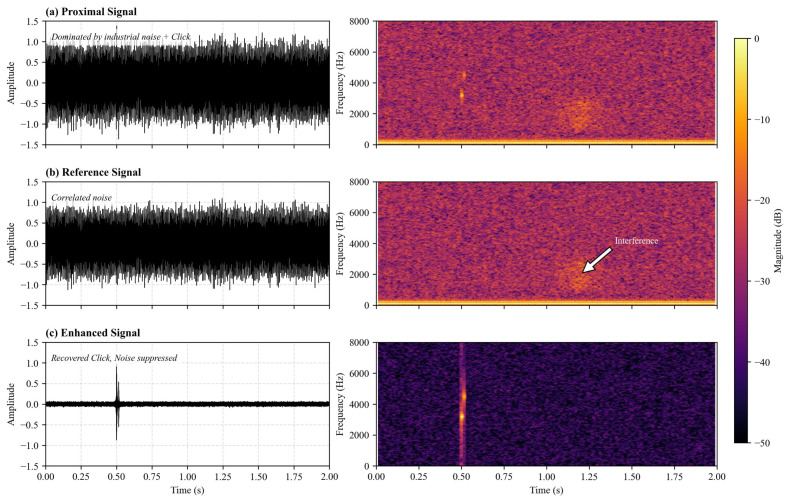
Spectro-temporal analysis at different processing stages. (**a**) Noisy proximal input dominated by industrial interference. (**b**) Ambient reference capturing the correlated noise floor. (**c**) Enhanced output by SC-DPM, showing restored transient morphology.

The fundamental premise of the proposed method is that reliable connector insertion detection depends critically on the high-fidelity recovery of short-duration transient acoustic signatures under extremely low signal-to-noise ratio (SNR) conditions. To qualitatively evaluate the reconstruction capability of the proposed spatially conditioned diffusion probabilistic model (SC-DPM), Figure 5 presents both time-domain waveforms and corresponding time–frequency representations at distinct processing stages.

#### 5.1.1. Proximal Signal Characteristics

As illustrated in Figure 5a, the source-proximal signal was severely dominated by broadband industrial noise and sporadic impulsive disturbances. In the time domain, the connector “click” event was virtually indistinguishable from background fluctuations. The spectrogram further reveals strong low-frequency mechanical noise and wideband interference that obscured the transient energy of the target event (spectro-temporal masking).

#### 5.1.2. Ambient Reference Signal Analysis

Figure 5b displays the signal captured by the ambient reference sensor. While this signal contained negligible information regarding the connector insertion (validating the physical isolation assumption), it exhibited a noise distribution that was highly correlated with the background component observed in the proximal signal. The highlighted interference region in the spectrogram confirms that dominant environmental noise sources were consistently captured, providing a physically meaningful spatial condition for noise suppression.

#### 5.1.3. Enhanced Signal via Spatially Conditioned Diffusion

Following processing by the proposed SC-DPM, the enhanced output in Figure 5c demonstrates substantial noise attenuation. In the time domain, the impulsive “click” event was clearly recovered, featuring a sharp onset and rapid decay. In the time–frequency domain, the reconstructed signal exhibited a concentrated vertical energy structure, indicating the successful restoration of transient spectral characteristics while actively suppressing broadband background noise.

**Figure 6:**
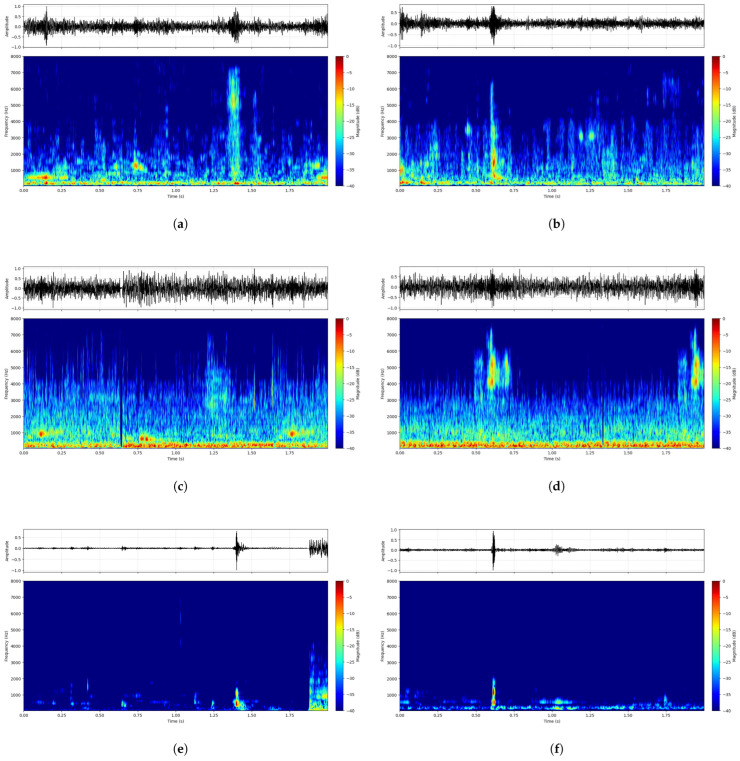
Representative reconstruction examples from the high-noise workshop dataset. (**a**,**b**) Input noisy signals. (**c**,**d**) Reference signals. (**e**,**f**) Enhanced outputs showing high-fidelity recovered transients.

To further validate the robustness of the reconstruction process, Figure 6 presents two representative insertion events from the high-noise workshop dataset. The top row (a–c) and bottom row (d–f) correspond to independent test samples.

- Input Noisy Signals (Figure 6a,d): Both examples demonstrate that the target transient was almost entirely submerged in non-stationary industrial noise, rendering discriminative detection infeasible.
- Ambient Reference Signals (Figure 6b,e): The reference channel captured consistent background noise patterns without introducing target-related artifacts, thereby serving as a reliable “negative” template.
- Enhanced Outputs (Figure 6c,f): The proposed method successfully reconstructed the transient insertion signatures in both cases, yielding clean and temporally localized events that closely resemble ideal baseline signals.

**Figure 7:**
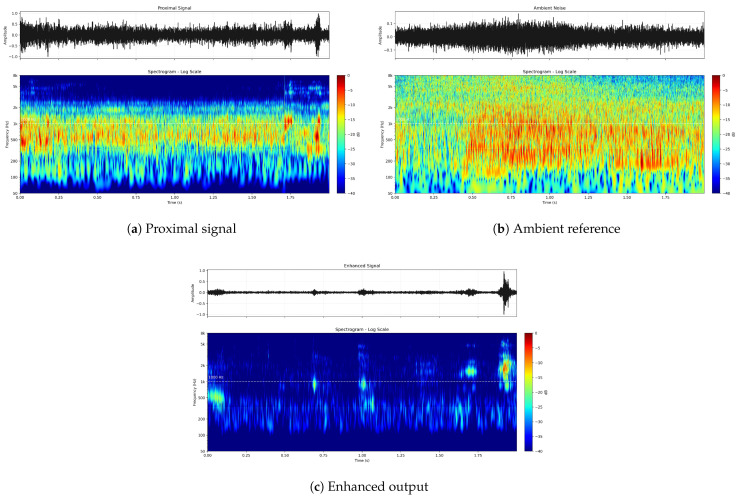
Log-scale spectrograms under overlapping broadband interference. (**a**) Proximal input containing strong disturbances, including interference extending above 1000Hz. (**b**) Ambient reference capturing correlated broadband environmental noise. (**c**) Enhanced output of SC-DPM, showing recovery of the transient click structure despite severe spectral masking.

To further examine the spectral overlap between the target transient and environmental interference, we additionally analyzed the proximal signal, the ambient reference signal, and the enhanced output using log-scale spectrograms, as shown in Figure 7. Compared with the linear-frequency visualizations in Figure 6, the log-scale representation made the low-frequency noise structure more visible and also showed that the interference was not confined below 1000Hz. In particular, both the proximal signal and the ambient reference contained substantial broadband disturbances extending into the frequency region relevant to the connector click. Even under this stronger spectral overlap, the enhanced output still exhibited a clearer and more concentrated transient pattern. This result indicates that the proposed SC-DPM can recover click-related characteristics even when the high-frequency band of interest is affected by strong environmental interference.

These visual results confirm that the proposed model did not function merely as a frequency-domain filter. Instead, it leveraged spatially correlated noise information to perform physically grounded manifold disentanglement, preserving the critical temporal morphology required for downstream classification.

### 5.2. Classification Accuracy

To quantitatively evaluate the impact of signal enhancement on event recognition, classification accuracy was assessed on two distinct datasets: OOD-Synthesized Noise (representing irregular outdoor interference) and Target Factory Domain (representing real-world assembly line noise). The comparative results are summarized in Table 1.

Key observations from the experimental data include the following:

1. Susceptibility to Domain Shift: In relatively controlled noise conditions, all learning-based methods achieved reasonable performance. However, in the challenging real factory environment, the accuracy of the baseline CRNN model degraded significantly to 69.4%, highlighting the severe impact of non-stationary industrial noise on standard deep learning architectures.
2. Baseline Performance: The SIMPF method [30], which utilizes simple pooling front-ends, demonstrated moderate robustness with an accuracy of 83.4%. Notably, our framework’s classification back-end alone (ablation) achieved only 76.8%, indicating that lightweight classifiers are insufficient to handle heavy industrial interference without proper signal enhancement.
3. Decisive Impact of Spatial Conditioning: The integration of the SC-DPM mechanism yielded a substantial performance leap. Specifically, when comparing the full model with the ablation version, the accuracy improved by approximately 16.5 percentage points (93.3–76.8%) in the factory environment. Furthermore, the proposed method outperformed the CRNN baseline by 23.9 percentage points, validating that explicitly modeling spatial noise correlation significantly enhances the discriminability between true insertion events and background disturbances.

These results quantitatively confirm that the proposed physics-aware generative demasking framework provides a critical advantage in complex acoustic environments.

### 5.3. Computational Cost and Efficiency Analysis

**Figure 8:**
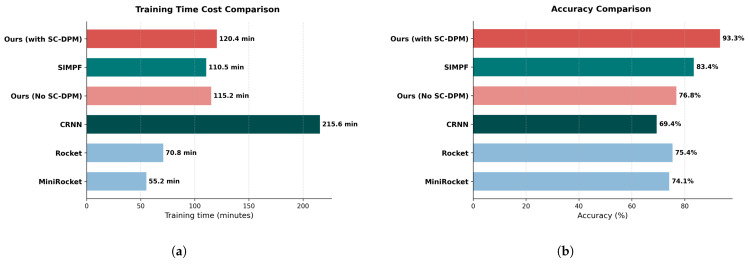
Cost–benefit analysis: comparison of training time (**a**) and classification accuracy (**b**) for different methods.

In addition to reconstruction fidelity and classification accuracy, practical industrial deployment requires high computational efficiency. Figure 8 illustrates the trade-off between training time and classification accuracy across different methods.

As shown in the left panel, lightweight time-series classifiers such as MiniRocket and Rocket achieved the shortest training times (55.2 min and 70.8 min, respectively), but their classification accuracy remained limited (74.1% and 75.4%, respectively), indicating insufficient robustness for safety-critical factory monitoring.

Conversely, deep learning–based methods incurred substantially higher computational costs. The CRNN model required the longest training time (215.6 min) while yielding the lowest accuracy (69.4%), demonstrating an unfavorable efficiency–performance ratio when operating on heavily noisy signals. SIMPF improved this balance (110.5 min, 83.4%), but its performance remained constrained by the lack of explicit spatial noise modeling.

For the proposed approach, a cost–benefit analysis revealed two critical findings:

- Marginal Overhead: Introducing the SC-DPM module increased the training time only slightly from 115.2 min (ablation) to 120.4 min (full), corresponding to a negligible 4.5% overhead.
- Significant Gain: This small additional cost yielded a massive return in performance, achieving the state-of-the-art accuracy of 93.3%. Compared with the ablated model, the accuracy gain exceeded 16 percentage points while maintaining nearly identical computational cost.

Overall, Figure 8 demonstrates that the proposed SC-DPM framework achieved an optimal Pareto frontier between efficiency and accuracy. By performing spatially conditioned diffusion-based noise suppression, the framework simplified the downstream classification task, avoiding reliance on excessively deep or computationally expensive models and making it highly suitable for real-time industrial deployment.

## 6. Conclusions

This work presented a physics-aware generative demasking framework designed to address the challenge of detecting transient acoustic events in extreme industrial noise environments. By leveraging a dual-sensor acquisition strategy, the proposed spatially-conditioned diffusion probabilistic model (SC-DPM) utilized the ambient reference signal as a dynamic physical condition, enabling the generative model to explicitly disentangle and reconstruct short-duration insertion signatures from heavily corrupted audio mixtures. Experimental validation on real-world datasets confirmed that this approach overcame the limitations of traditional “blind” denoising methods, achieving a classification accuracy of 93.3% and demonstrating superior robustness against non-stationary interference. Crucially, we demonstrated that the proposed “restore-then-classify” paradigm—coupled with efficient causal random kernel feature extraction—successfully bridges the gap between the high fidelity of generative models and the low-latency requirements of industrial deployment. These findings suggest that spatially conditioned generative restoration offers a scalable and physically grounded solution for zero-defect manufacturing, extending the frontiers of acoustic sensing in complex, noise-intensive scenarios.

One limitation of the present study is that it did not fully examine more challenging interference cases, especially highly similar overlapping click-like sounds generated by nearby operations. Such transient interference may share similar temporal and spectral characteristics with the target insertion event, making reliable discrimination more difficult. In future work, this limitation could be addressed by extending the dataset to more complex interference scenarios and by introducing richer sensing strategies, such as multi-microphone spatial localization or multi-modal information, to improve the separation of target clicks from adjacent transient disturbances.

## Figures and Tables

**Table 1:** Classification accuracy comparison on different datasets.

Model	OOD-Synthesized Noise	Target Factory Domain
CRNN Baseline	81.9%	69.4%
SIMPF [30]	88.1%	83.4%
Ours (Ablation: No SC-DPM)	85.3%	76.8%
Ours (Full: with SC-DPM)	94.8%	93.3%

## Data Availability

The raw data supporting the conclusions of this article will be made available by the authors on request.
